# Vaccine-Linked Chemotherapy Approach: Additive Effects of Combining the *Listeria monocytogenes*-Based Vaccine Lm3Dx_NcSAG1 With the Bumped Kinase Inhibitor BKI-1748 Against *Neospora caninum* Infection in Mice

**DOI:** 10.3389/fvets.2022.901056

**Published:** 2022-06-27

**Authors:** Dennis Imhof, William Robert Pownall, Carling Schlange, Camille Monney, Luis-Miguel Ortega-Mora, Kayode K. Ojo, Wesley C. Van Voorhis, Anna Oevermann, Andrew Hemphill

**Affiliations:** ^1^Department of Infectious Diseases and Pathobiology, Institute of Parasitology, Vetsuisse Faculty, University of Bern, Bern, Switzerland; ^2^Graduate School for Cellular and Biomedical Sciences, University of Bern, Bern, Switzerland; ^3^Division of Small Animal Surgery, Department of Clinical Veterinary Science, Vetsuisse Faculty, University of Bern, Bern, Switzerland; ^4^Department of Clinical Research and Veterinary Public Health, Division of Neurological Sciences, DCR-VPH, Vetsuisse Faculty, University of Bern, Bern, Switzerland; ^5^SALUVET, Animal Health Department, Faculty of Veterinary Sciences, Complutense University of Madrid, Madrid, Spain; ^6^Center for Emerging and Re-Emerging Infectious Diseases (CERID), Division of Allergy and Infectious Diseases, Department of Medicine, University of Washington, Seattle, WA, United States

**Keywords:** neosporosis, vaccine, vaccine-linked chemotherapy, bumped kinase inhibitor, pregnancy, vertical transmission

## Abstract

The apicomplexan parasite *Neospora (N.) caninum* causes neosporosis in numerous host species. There is no marketed vaccine and no licensed drug for the prevention and/or treatment of neosporosis. Vaccine development against this parasite has encountered significant obstacles, probably due to pregnancy-induced immunomodulation hampering efficacy, which has stimulated the search for potential drug therapies that could be applied to limit the effects of neosporosis in dams as well as in offspring. We here investigated, in a pregnant neosporosis mouse model, the safety and efficacy of a combined vaccination-drug treatment approach. Mice were vaccinated intramuscularly with 1 × 10^7^ CFU of our recently generated *Listeria (L.) monocytogenes* vaccine vector expressing the major *N. caninum* tachyzoite surface antigen NcSAG1 (Lm3Dx_SAG1). Following mating and experimental subcutaneous infection with 1 × 10^5^ *N. caninum* (NcSpain-7) tachyzoites on day 7 of pregnancy, drug treatments were initiated using the bumped kinase inhibitor BKI-1748 at 20 mg/kg/day for 5 days. In parallel, other experimental groups were either just vaccinated or only treated. Dams and offspring were followed-up until day 25 *post-partum*, after which all mice were euthanized. None of the treatments induced adverse effects and neither of the treatments affected fertility or litter sizes. Cerebral infection in dams as assessed by real-time PCR was significantly reduced in the vaccinated and BKI-1748 treated groups, but was not reduced significantly in the group receiving the combination. However, in non-pregnant mice, all three treatment groups exhibited significantly reduced parasite burdens. Both, vaccination as well BKI-1748 as single treatment increased pup survival to 44 and 48%, respectively, while the combination treatment led to survival of 86% of all pups. Vertical transmission in the combination group was 23% compared to 46 and 50% in the groups receiving only BKI-treatment or the vaccine, respectively. In the dams, IgG titers were significantly reduced in all treatment groups compared to the untreated control, while in non-pregnant mice, IgG titers were reduced only in the group receiving the vaccine. Overall, vaccine-linked chemotherapy was more efficacious than vaccination or drug treatment alone and should be considered for further evaluation in a more relevant experimental model.

## Introduction

*Neospora (N.) caninum* is an obligate intracellular apicomplexan parasite and the causative agent of neosporosis in cattle, dogs and many other animal species (1). Neosporosis has a major impact on cattle industry, with economic losses of around 1.3 billion US dollar per year due to *N. caninum*-induced abortions, stillbirth, and birth of weak offspring (2, 3). Additionally, *N. caninum* infects small ruminants including sheep and goats causing abortion, and has been detected in many other domestic and wildlife animal species, but not in humans (1). Three distinct infectious stages contribute to the life cycle of *N. caninum*: (i) environmentally resistant oocysts are formed in the intestine of the canid definitive host excreted with the feces, and sporozoites are formed in the environment; (ii) rapidly proliferating tachyzoites, which cause acute disease in the intermediate hosts including abortion, encephalomyelitis and myositis; and (iii) slowly replicating bradyzoites that form tissue cysts which can persist for years in the intermediate hosts without eliciting an inflammatory immune response (1, 4–6). Infection occurs either through the oral route (horizontal transmission *via* tissue cysts or oocysts), or through vertical transmission with tachyzoites crossing the placental barrier and infecting the fetus, the latter being the most frequently encountered infection mode (1, 2, 6, 7). Upon infection, clinical signs are absent to mild in immunocompetent animals. However, in dams infected during gestation, either by primary infection or by reactivation of a latent infection, tachyzoites can reach the placenta and infect the fetus by exogenous or endogenous transplacental transmission, respectively. This can result in abortion, birth of weak calves exhibiting neurological clinical signs, or persistently infected but clinically healthy calves that transmit the parasite to the next generation (6–10). Despite its economic importance as a global veterinary health problem, commercially available vaccines or drugs for the prevention or treatment of neosporosis are not available on the market (2, 11).

Vaccination has been proposed to be the most promising and cost-effective means of neosporosis control. A large panel of potential vaccine candidates for the prevention of *N. caninum* infection has been investigated, including subunit vaccines containing recombinant antigens or fractionated parasites extracts, DNA-vaccines, or antigens expressed in viral vaccine vectors [for review see (1)]. In addition, a plethora of adjuvant formulations have been investigated (12). Mice studies have shown that considerable protective effects against experimental infection could be induced in non-pregnant rodents, but in most cases, protection was lost upon pregnancy due to pregnancy-associated immune modulation, and the same vaccine formulations did not protect offspring from vertical transmission (11). Studies carried out in cattle and sheep models employed killed tachyzoite extracts emulsified in various solvents, naturally attenuated live vaccine strains, as well as recombinant antigens such as SAG1, HSP20 and GRA7 formulated in ISCOM or in oligomannose microsomes (13). Current evidence clearly suggests that attenuated live vaccine strains represent the most promising option for neosporosis control (14).

*Listeria (L.) monocytogenes* is a gram-positive facultative intracellular bacterium, which upon infection induces strong cellular and innate immune responses. More recently, attenuated *Listeria*-based vaccine vectors have gained increasing importance in clinical trials for the prevention of infectious diseases and cancer therapy (15, 16). We reported earlier on the deletion of the three essential virulence genes *actA, inlA*, and *inlB* of our *L. monocytogenes* strain JF5203, resulting in the strongly attenuated vaccine strain Lm3Dx (17). By insertion of the gene coding for the highly immunogenic *N. caninum* surface antigen NcSAG1, the vaccine strain Lm3Dx_NcSAG1 was generated. Non-pregnant BALB/c mice, which were immunized three times with Lm3Dx_NcSAG1 developed a Th1-biased immune response against NcSAG1 (17). In addition, vaccination of mice with Lm3Dx_NcSAG1 was safe and efficacious. Survival rates of pups born from dams vaccinated thrice at 2-week intervals with 1 × 10^7^ CFU of Lm3Dx_NcSAG1 and infected with *N. caninum* tachyzoites during pregnancy were approximately 70%, while no survivors were found in non-vaccinated controls. Furthermore, cytokine measurements done at the endpoint showed that the attenuated mutant vaccine vector induced a balanced Th1/ Th2 response (18).

Another potential option to control neosporosis is drug treatment. Ideally, such drugs should be able to cross the placental and brain barriers, not induce adverse side effects during pregnancy, successfully prevent vertical transmission of *N. caninum* tachyzoites and reduce cerebral parasite burden in dams and offspring (19). Bumped-kinase inhibitors (BKIs) belong to a group of compounds, which selectively target the calcium-dependent protein kinase 1 (CDPK1), an excellent drug target that is expressed in apicomplexan parasites and plants but not in mammals (20). CDPK1 plays an important role in gliding motility, invasion, exocytosis, and egress of the parasite (21–23). Several BKIs have shown promising efficacy against *N. caninum in vitro* and in experimentally infected pregnant mice (23–25), and some BKIs have been advanced to the pregnant sheep infection model (24, 26, 27). BKI-1748 is a recently introduced 5-aminopyrazol-4-carboxamide-based compound with an *in vitro* EC_50_ of 165 nM against *N. caninum* and no impairment of human foreskin fibroblasts (HFF) viability up to 20 μM (28, 29). BKI-1748 did not affect zebrafish embryo development at 10 μM (30), and cardiovascular safety studies in rats and dogs showed only small abnormalities at 18 μM and above (31). BKI-1748 treatment in pregnant mice at 20 mg/kg/day for five consecutive days did not interfere with pregnancy outcome and led to significant inhibition of vertical transmission and increased pup survival in *N. caninum* infected mice (28, 29).

To improve the preventive and therapeutic treatments for neosporosis, new avenues should be considered, one of which could be a vaccine-linked chemotherapy approach. This approach relies on a combination between vaccination and treatment, in which immunity is generated with a vaccine and is supplemented by drug treatment. This approach, originally developed and applied for cancer treatments, has not been widely used for the preventive treatment of parasitic infections. One example is East coast fever in cattle caused by the apicomplexan *Theileria parva*. To protect against *Theileria* infection, cattle are vaccinated with live sporozoites together with a drug that affects parasite viability such as tetracycline, or alternatively buparvaquone (32, 33). Combination of vaccination using parasite antigen extracts or defined recombinant antigens with chemotherapy was also assessed for the treatment of canine visceral leishmaniosis (34). An example is the study on BALB/c mice infected with *Leishmania donovani* (35) or hamsters infected with *L. infantum* causing visceral leishmaniosis (36). Combining benznidazole treatment in *Trypanosoma cruzi* infected mice with a recombinant vaccine candidate formulated with a synthetic TLR4 receptor agonist led to significant improvement of treatment efficacy, significantly reduced cardiac inflammation, fibrosis, and improved survival of infected mice (37, 38).

We here present results of a study investigating the effect of vaccination with Lm3Dx_SAG1 followed-up by BKI-1748 treatment in pregnant BALB/c mice infected with *N. caninum*. We show that application of this combined approach leads to an additive effect in the prevention of vertical transmission as well as an improvement of offspring survival compared to vaccination or drug treatment alone.

## Materials and Methods

### Cell Culture Media, Biochemicals, and Compound Preparation

If not stated otherwise, all culture media were purchased from Gibco-BRL (Zürich, Switzerland), and biochemicals were acquired from Sigma (St. Louis, MO, USA). BKI-1748 was originally synthesized in the Department of Biochemistry of the University of Washington, USA (39) and scaled up by WuXi Apptec Inc., Wuhan, China to >98% purity by LC/MS-MS and NMR and shipped as powder. For *in vitro* studies, stock solutions of 20 mM were prepared in dimethyl-sulfoxide (DMSO) and stored at −20°C. For *in vivo* experiments, BKI-1748 was suspended in sterile corn oil prior to administration of mice by oral gavage.

### Culture of Host Cells, Maintenance of Parasites *in vitro*, and Generation of Lm3Dx_NcSAG1

Human foreskin fibroblasts (HFF; ATCC^®^ SCRC-1041^TM^) were maintained in Dulbecco's modified Eagles' medium (DMEM) including 10% fetal calf serum (FCS) and 1% antibiotics/antimycotics at 37°C, 5% CO_2_ in T25, T75 or T125 cell culture flasks (Sarstedt, Sevelen, Switzerland). BALB/c dermal fibroblasts (DF; CELLNTEC Advanced Cell systems AG, Bern, Switzerland) were cultured under the same conditions as described for HFF. *N. caninum* NcSpain-7 tachyzoites were cultured in HFF for several passages as described before and transferred to BALB/c DF prior to infection of mice (40). The attenuated mutant vaccine vector Lm3Dx_NcSAG1 was engineered and fosfomycin minimal inhibitory concentration (MIC) as well as genetic stability of Lm3Dx_NcSAG1 were evaluated as previously described in detail in (17). In short, the Lm3DX mutant strain was generated from *L. monocytogenes* strain JF5203 (NCBI Reference Sequence: NZ_LT985474.1) by homologous recombination with either pMAD or pHOSS1 plasmid derivates (17). The JF5203DactA mutant was used as parental mutant to engineer the vaccine vector JF5203DactA/inlA/inlB/fosX (Lm3Dx) by sequential in-frame deletion of *inlA/inlB* and *fosX* genes by homologous recombination with the respective pMAD and pHOSS1 plasmid derivates (17). Gene deletions were confirmed by DNA sequencing of the intermediate mutants and by full genome sequencing of the final vaccine vector strain (17). Finally, Lm3Dx was transformed with the plasmid pMAD_NactA100AA_SAG1 (17) to yield Lm3Dx_SAG1 expressing the *sag1* gene fused to the 300 first nucleotides of *actA* under the control of the *actA* promotor.

### Ethics Statement

The animal protocols were approved by the Animal Welfare Committee of the Canton of Bern (License BE 113/19). Female and male BALB/c mice, 6-week-old, were ordered from a commercial breeder (Charles River, Sulzberg, Germany) and were maintained in a common room under controlled temperature and a 14-h light/10-h dark cycle according to guidelines of the Animal Welfare Legislation of the Swiss Veterinary Office. Mice were housed in the animal facility for 2 weeks prior the start of the vaccine-linked chemotherapy experiment for adaptation to the environmental conditions.

### Efficacy Evaluation, Experimental Set-Up, Clinical Monitoring and Sample Collection

**Figure 1:**
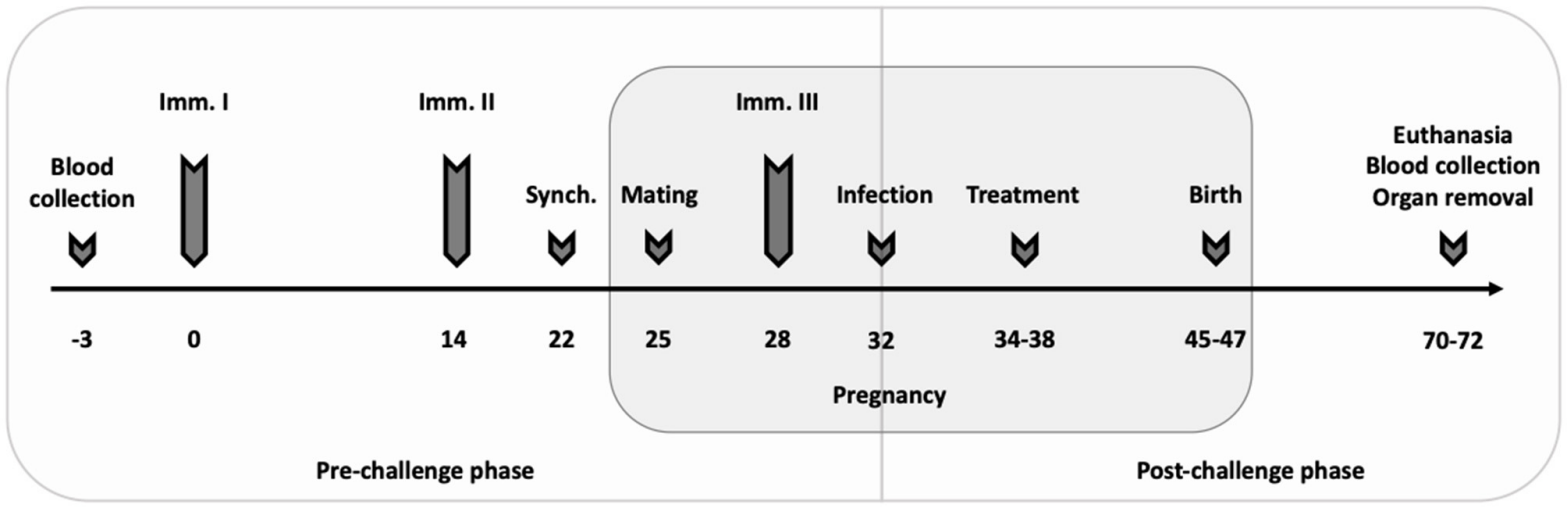
Experimental setup. Mice were vaccinated three times at 2-week intervals (Imm. I–III) with the third immunization 3 days after mating. Mice were oestrus-synchronized (Synch.) 8 days after the second vaccination before two females and one male were housed together for mating. Seven days post-mating, all mice except the negative control (C– group) were infected with a sublethal dose of 1 × 10^5^ NcSpain-7 tachyzoites. The C– group was inoculated with BALB/c DF. Treatment with 20 mg/kg BKI-1748 dissolved in corn oil was applied 2 days post-infection by oral gavage for five consecutive days. Mice were daily monitored for clinical signs, and neonatal and postnatal mortality were recorded. All adult mice and pups were euthanized between day 70–72, corresponding to 25 days p.p.

Sixty-eight female and 34 male BALB/c mice were used for the vaccine-linked chemotherapy study. After 2 weeks of acclimatization, female mice were randomly distributed into five experimental groups (14 mice per experimental group, 12 mice in negative control group; two females per cage): [1] 1 × 10^7^ CFU Lm3Dx_NcSAG1 + 20 mg/kg BKI-1748 + 1 × 10^5^ NcSpain-7 tachyzoites (combination); [2] 1 × 10^7^ CFU Lm3Dx_NcSAG1 + 1 × 10^5^ NcSpain-7 tachyzoites (vaccine control); [3] 20 mg/kg BKI-1748 + 1 × 10^5^ NcSpain-7 tachyzoites (treatment control); [4] 1 × 10^7^ CFU Lm3Dx (empty vector) + 1 × 10^5^ NcSpain-7 tachyzoites (positive control, C+); [5] PBS + BALB/c dermal fibroblasts (negative control, C-). The experimental setup with immunization protocol, mating, infection, blood and organ collection as well as euthanasia is illustrated in Figure 1. Following the collection of pre-immunization blood at day−3, mice in the combination and the vaccine control groups were immunized intramuscularly in the thigh musculature twice with a 2-weeks interval on days 0 and 14 with 1 × 10^7^ CFU Lm3Dx_NcSAG1 (17, 18), the positive control group was inoculated with 1 × 10^7^ Lm3Dx (empty vector), the other groups received PBS. Eight days after the second immunization (day 22), mice were oestrus-synchronized during 3 days by the Whitten effect (41), and were subsequently mated for 3 days. Males were separated from females on day 26 and the third vaccination was applied on day 28, 4 days prior to challenge infection on day 32 with a sublethal dose (1 × 10^5^ tachyzoites) of the highly virulent *N. caninum* strain NcSpain-7 (18, 42). Mice of the negative control (C-) received BALB/c DF only. Two days later, the mice in the combination and in the treatment control group received 20 mg/kg BKI-1748 emulsified in corn oil for five consecutive days (day 34–38) by oral gavage (28), the other groups received corn oil only.

After challenge, mice were observed daily and were weighted every third day to confirm pregnancy and/or to identify potential abortions. At day 18 post-mating (day 43), pregnant mice were separated and transferred into single cages, while non-pregnant mice were maintained in groups of three animals. Pregnant mice gave birth between day 20 and 22 of pregnancy (day 45–47). Data on fertility, litter size, clinical signs, neonatal and postnatal mortality were collected daily. Twenty-five days post-partum (p.p.), all mice and pups were euthanized using isoflurane and CO_2_. Blood was taken prior to the first vaccination from the tail vein and at the endpoint by cardiac puncture. Serum was extracted afterwards by centrifugation for 12 min at 1,200 × g. Brain hemispheres were sampled aseptically, and all samples were stored at −20°C until further use.

### Evaluation of Cerebral Parasite Load by Real-Time (RT)-qPCR

The cerebral parasite load was quantified in all adult mice and surviving pups by RT-qPCR designed for *N. caninum* (43, 44). DNA purification was conducted with the NucleoSpin DNA RapidLyse Kit (Macherey-Nagel, Oesingen, Switzerland) according to the manufacturer's instruction. Concentration of DNA was quantified by using the QuantiFluor double-stranded DNA system (Promega, Madison, WI, USA). TaqMan probe-based RT-qPCR was performed in a CFX96 qPCR instrument (Bio-Rad Laboratories AG, Cressier, Switzerland) to quantify *N. caninum* DNA. CFX manager software version 1.6 was used for the analysis of the PCR results. RT-qPCR is targeted to the repetitive genomic sequence NC5 of *N. caninum* (43). The reaction mixture (10 μl per reaction) contains 5 μl of 2× Mastermix (SensiFAST™ Probe NO-ROX Kit; Bioline Meridian Lifescience, Memphis, TN, USA), 500 nM forward primer Np21plus (5′-CCCAGTGCGTCCAATCCTGTAAC-3′) and reverse primer Np6plus (5′-CTCGCCAGTCAACCTACGTCTTCT-3′) (43), 100 nM of detection probe NC5–1 (5′-*FAM*-CACGTATCCCACCTCTCACCGCTACCA-*BHQ-1*-3′) (44) and 5 ng of sample DNA. Additionally, 300 nM dUTP (supplementary to dTTP included in the 2× Mastermix) and one unit of heat-labile Uracil DNA Glycosylase (UDG; both from Bioline Meridian Lifesciences) were included in the reaction mixture to remove eventual carry-over contaminations from previous reactions as described earlier (45). For UDG-mediated decontaminations, the temperature profile included an initial 10 min incubation at 40°C followed by a 5 min denaturation period at 95°C. Subsequently, DNA amplification was achieved during 50 cycles of 10 s at 95°C and 30 s at 60°C. After each cycle, light emission by the fluorophore was measured at 60°C. Brain samples from non-pregnant and pregnant mice were tested in duplicates, while pup brains were measured in single values (18). As external quantification standards, samples containing DNA equivalents from approximately 10,000, 1,000, 100, 10 *N. caninum* tachyzoites were included.

### Analysis of Humoral Immune Responses by Enzyme-Linked Immunosorbent Assay

IgG antibody titers against *N. caninum* crude extract were assessed by ELISA as described previously (18, 46, 47). In brief, 96-well plates were coated over night at 4°C with 200 ng of *N. caninum* crude extract in 100 μl of coating buffer per well. After three washes, plates were blocked with 1% bovine serum albumin in wash buffer [PBS with 0·05% Tween-20 (v/v)] and serum samples incubated at 1:100. After three washes, plates were incubated with either goat anti-mouse IgG1 or IgG2aconjugated with alkaline phosphatase (AP; SouthernBiotech, Birmingham, USA). The reaction was developed with AP substrate and absorbance values as optical density (OD) at 405 nm were read in a tunable microplate reader (EnSpire™ 2300 Multilabel Reader, Switzerland). To compare OD values between samples, the same positive and negative serum controls were added in each plate and OD values for each sample were converted into a relative index per cent (RIPC) using the following formula: RIPC = (OD405 sample– OD405 negative control)/(OD405 positive control – OD405 negative control) × 100.

### Statistics

Cerebral parasite burdens and IgG antibody titers were compared between groups by the non-parametric Kruskal–Wallis test including Dunn's multiple comparison correction. If statistical differences were detected, a Mann–Whitney *U*-test was subsequently applied comparing only two groups with each other. Neonatal and postnatal mortality rates as well as vertical transmission rates were analyzed between the three experimental groups (combination, vaccine, treatment) and the positive control group by Chi-square (and Fisher's exact) test. The pup mortality along time was compared by plotting survival events at each time point in Kaplan-Meier graphs and survival curves were compared by the Log-rank (Mantel-Cox) test. Statistical analysis was performed using Graphpad Prism version 9.3.1 for macOS (GraphPad Software, La Jolla, CA, USA, www.graphpad.com).

## Results

### Safety and Efficacy Evaluation of Lm3Dx_NcSAG1 in Combination With BKI-1748 in Non-pregnant Mice, Dams and Pups

Results of the experiments are summarized in Table 1. Safety characteristics of the attenuated mutant strain Lm3Dx_NcSAG1 were previously evaluated (17). The combination therapy of 1 × 10^7^ CFU Lm3Dx_NcSAG1 and BKI-1748 treatment at 20 mg/kg did not interfere with pregnancy outcomes. Reproductive parameters such as fertility rates and litter size were not affected. Similar observations regarding reproduction and fertility rates were made in the vaccine and treatment groups as well as in both control groups.

**Table 1:** Summary of the serological evaluation, cerebral parasite burden, litter size, and neonatal and postnatal mortality rates of this experiment.

**Vaccine**	**Compound**	**Challenge** **dose** **NcSp-7 tachyzoites**	**Sero-pos. all mice**	**PCR-pos non-preg**	**PCR-pos dams**	**No pups/** **dams (**∅**)**	**Neonatal mortality^**a**^** **(%)**	**Postnatal mortality^**b**^** **(%)**	**PCR-pos pups** **(%)**
Lm3Dx_NcSAG1 1 ×10^7^ CFU	BKI-1748 20 mg/kg	1 ×10^5^	14/14	2/8^1^	3/6	37/6 (6.2)	2/37 (5.4)	5/35 (14)^4^	8/35 (23)^4^
Lm3Dx_NcSAG1 1 ×10^7^ CFU	Corn oil	1 ×10^5^	14/14	1/7^2^	5/7	44/7 (6.3)	0/44 (0)^3^	21/44 (48)^4^	22/44 (50)^4^
PBS	BKI-1748 20 mg/kg	1 ×10^5^	14/14	5/8	3/6	41/6 (6.8)	2/41 (4.9)	17/39 (44)^4^	18/39 (46)^4^
Lm3Dx-no insert 1 ×10^7^ CFU	Corn oil	1 ×10^5^	14/14	8/8	6/6	39/6 (6.5)	6/39 (15.4)	33/33 (100)	33/33 (100)
PBS	Corn oil	1 ×10^5^ DF^c^	0/12	0/5	0/7	41/7 (5.9)	2/41 (4.9)	0/39 (0)	0/39 (0)

*The combination group was immunized three times with 1 × 10^7^ CFU Lm3Dx_NcSAG1, challenged with 1 × 10^5^ Neospora caninum NcSp-7 tachyzoites, and subsequently treated with 20 mg/kg BKI-1748 in corn oil for 5 days. Mice in the vaccine control group were vaccinated, challenged, and were given corn oil only, while mice in the treatment control group were inoculated PBS, but were challenged and subsequently treated with BKI-1748. The positive control group was immunized with 1 × 10^7^ CFU of the empty vector Lm3Dx and was also challenged. Mice in the negative control group were neither vaccinated, inoculated with BALB/c dermal fibroblasts (DF) and given corn oil only. After euthanasia at day 25 p.p., the cerebral parasite load of non-pregnant mice, dams and surviving pups was analyzed by quantitative RT-qPCR. Pups which died before the end of the study were considered as N. caninum PCR-positive. Animals from the experimental groups were compared with the positive control group by the Chi-square (and Fisher's exact) test*.

^1^Indicates **P < 0.007.

^2^**P < 0.0014.

^3^**P < 0.0086.

^4^****P < 0.0001.

*^a^Number of pups born dead or died within the first 2 days post-partum*.

*^b^Number of pups that died between day 3 and 25 post-partum*.

*^c^DF = BALB/c dermal fibroblasts*.

All mice in the vaccinated and treated group showed no clinical signs of neosporosis until the end of the study and surviving pups were in good health conditions. Similar observations were made for adult mice and pups in the treatment control group, while 1 out of 7 pregnant mice that were vaccinated three times with 1 × 10^7^ Lm3Dx_NcSAG1 exhibited moderate neurological signs at the end of the study. Neurological signs developed after birth of pups and were compatible with neosporosis, while non-pregnant mice and surviving pups in the vaccine control group remained healthy until the end of the experiment. One out of six dams in the positive control group, where mice had been inoculated with the empty vector (Lm3Dx), showed strong signs of paralysis and ruffled fur. This dam had eaten most of the pups' body after they died and clinical signs developed after birth of the pups. In the same group, one non-pregnant mouse had to be euthanized before the end of the study due to neurological clinical symptoms and coordination issues.

**Figure 2:**
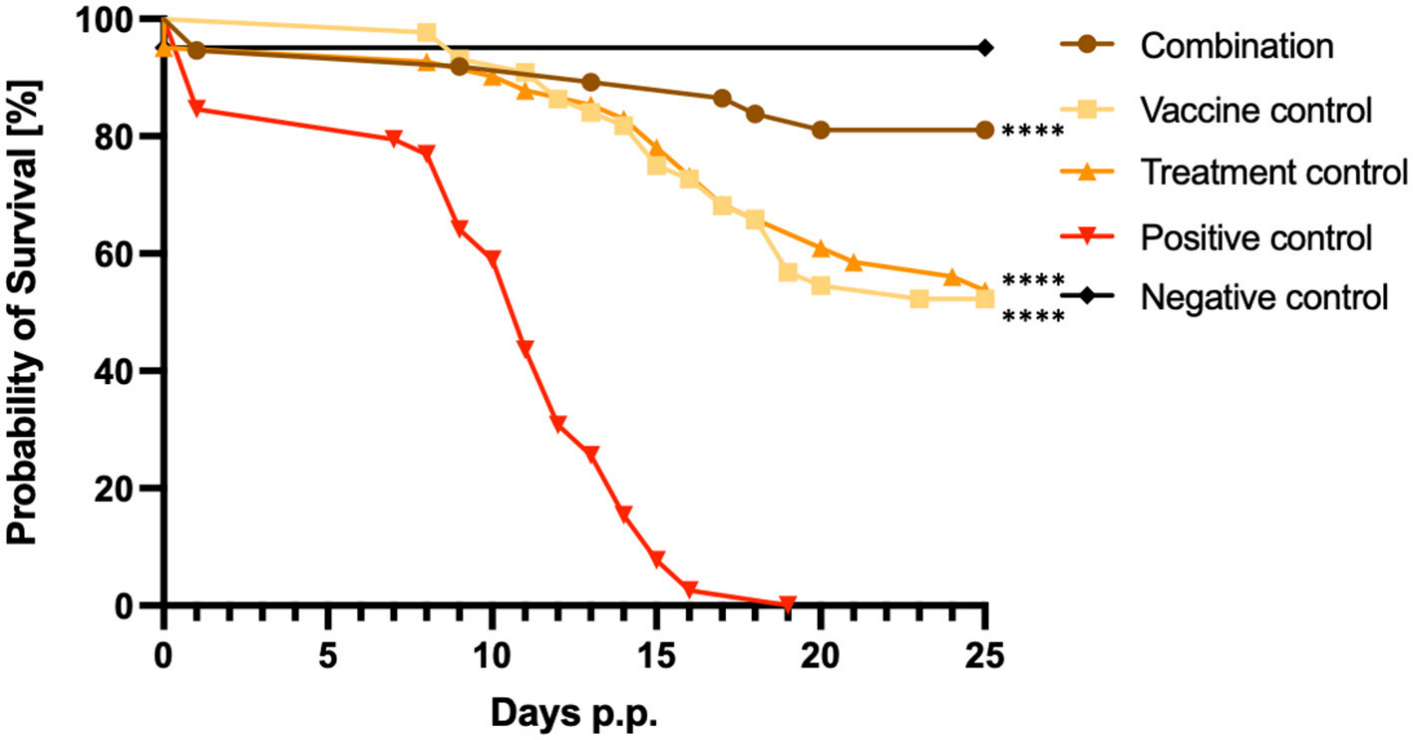
Kaplan–Meier survival graphs of the different treatment groups. Survival rates of pups were plotted daily in Kaplan–Meier graphs and individual curves were analyzed by the Log-rank (Mantel-Cox) test. Significant differences were calculated by comparing the combination, the vaccine control and the treatment control curves with the survival curves of the C+ control group (****P < *0.0001*).

Vaccination in combination with BKI-1748 treatment resulted in 86% of pup survival, while in the vaccine and treatment control groups only 52% and 56% of the pups survived until 25 days p.p., respectively (Figure 2). In the positive control group, all pups were dead by day 19 p.p. corresponding to 6 days before the study was terminated, while all except two pups survived in the negative control group inoculated with dermal fibroblasts. These two pups died in the neonatal phase and the reason for that could be birth complications (Figure 2). Highly significant differences were achieved by comparing the combination, the vaccine and the treatment survival curves with the curve from the positive control group. [^****^P < *0.0001*, Log-rank (Mantel-Cox) test].

### Assessment of Cerebral Parasite Burden

**Figure 3:**
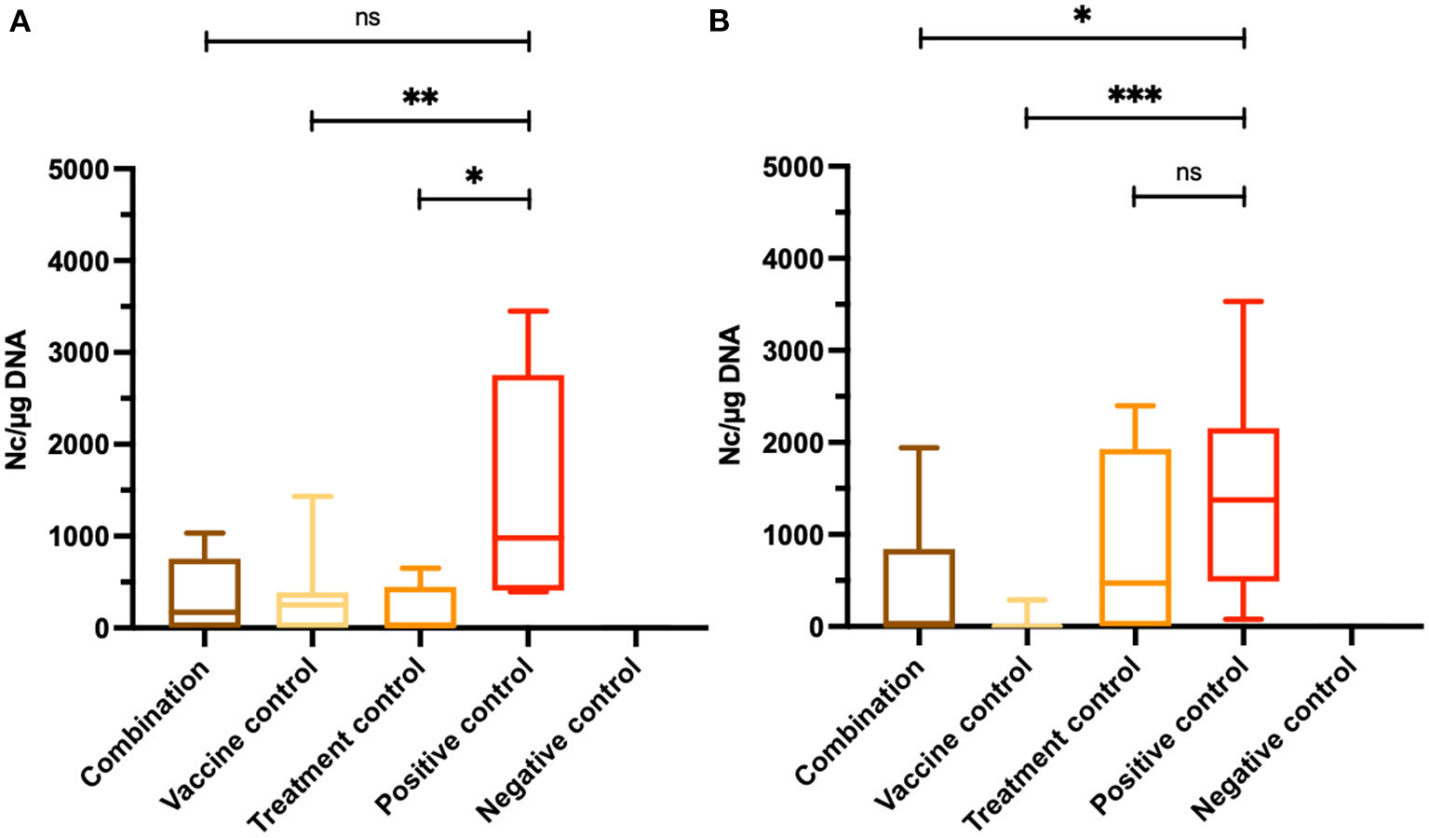
Assessment of cerebral parasite burden. Results for dams **(A)** and non-pregnant mice **(B)** were obtained by quantitative RT-qPCR. Brain samples of adult mice were collected aseptically after euthanasia. Values are shown as box plots. In dams, no statistically significant reduction of cerebral parasite burden was achieved between the combination group and the positive control, but parasite loads were significantly reduced comparing the vaccine and the treatment control groups with the C+ group (**P < *0.0082*, *P < *0.0152*, Mann–Whitney *U*-test). In contrary, parasite burden of non-pregnant mice from the combination group were significantly decreased comparing the vaccine control and the positive control, as well as between the treatment control group and the C+ group (*P < *0.0154*, ***P < *0.0006*, Mann–Whitney *U*-test). No significant differences were measured between the treatment control group and the positive control group.

Three out of six brain samples from dams in the combination group, five out of seven brains in the vaccine control group and three out of six brains in the treatment control group were tested PCR-positive for *N. caninum*, while all brain samples in the positive control group were *N. caninum* PCR-positive. All mice in the negative control group were tested negative by RT-qPCR (Table 1). Vaccination and treatment alone had a protective effect causing reduction of protozoal load in the brain of non-pregnant and pregnant animals compared to the C^+^ group (^**^P < *0.0082*, ^*^P < *0.0152*, Mann–Whitney *U*-test; Figure 3A), However the combination did not cause further reduction. In the combination group, variation was higher and therefore, the reductive effect compared to the positive control did not reach significance.

In non-pregnant mice, only two out of eight animals from the combination group were tested *N. caninum* PCR-positive, while in one out of seven animals of the vaccine control group *N. caninum* DNA was detected in brain samples. In the treatment control group, five out of eight mice were PCR-positive. *Neospora caninum* DNA was found in all brain samples from non-pregnant mice of the positive control group (Table 1). The cerebral parasite load of non-pregnant mice in the combination group was significantly reduced compared to the C^+^ group (^*^P < *0.0154*, Mann–Whitney *U*-test). Almost all brain samples from non-pregnant mice of the vaccine control group were *N. caninum* PCR-negative resulting in a highly significant reduction of cerebral parasite burden compared to the positive control group (^***^P < *0.0006*, Mann–Whitney *U*-test). No significant differences were measured between the treatment control group and the C^+^ group (Figure 3B).

Vertical transmission was strongly reduced in dams that received the vaccination in combination with the BKI-1748 treatment, with 77% of pups testing *N. caninum* PCR-negative. Fifty percentage of pups were tested PCR-negative from dams of the vaccine only group and 54% of pups from the BKI-1748 only treatment group. Vertical transmission was 100% in the positive control group and no *N. caninum* DNA was detected in the negative control group (Table 1).

### Humoral Immune Responses

**Figure 4:**
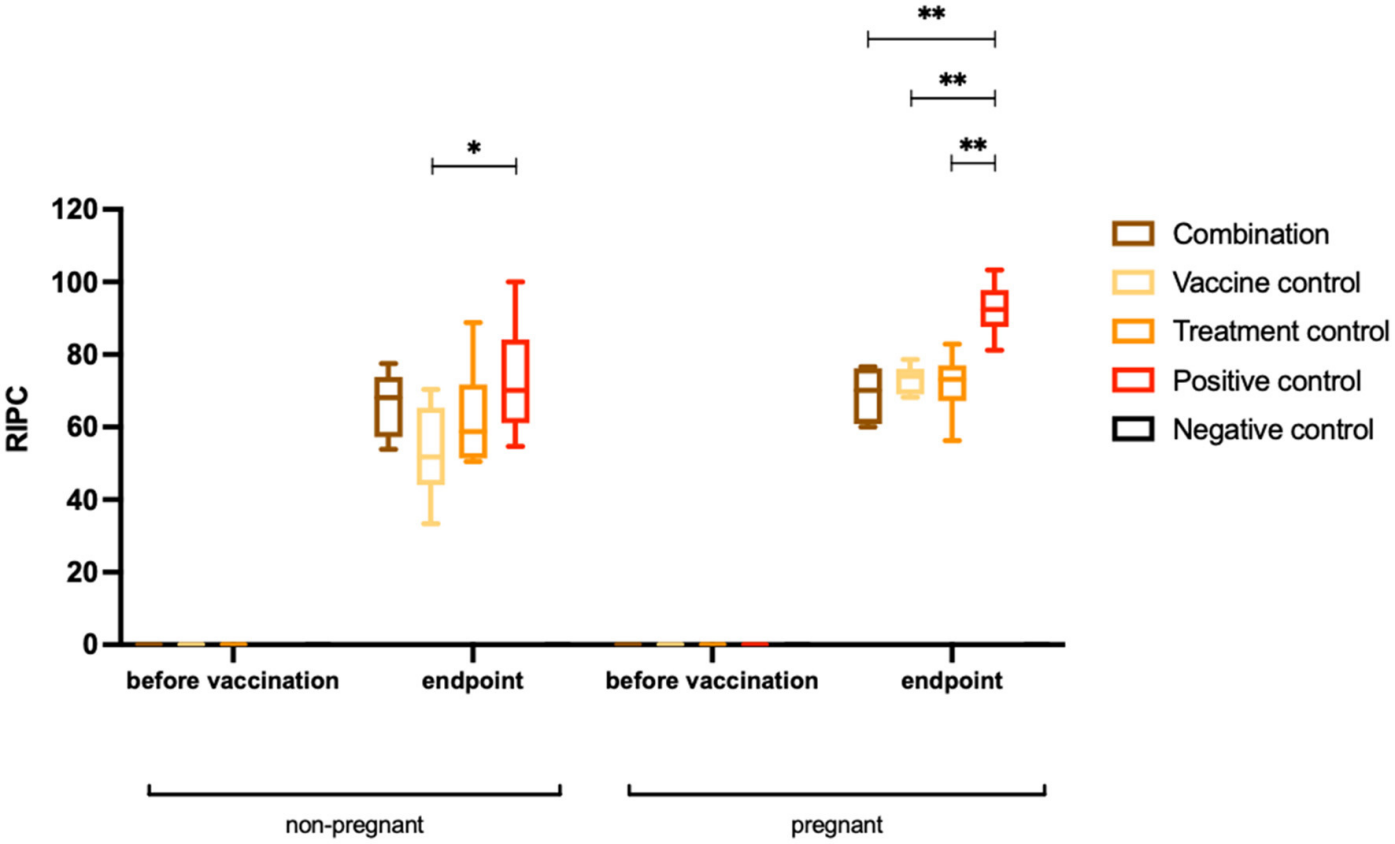
Humoral immune responses (IgG titers) against *Neospora caninum* extracts. Results are shown as box plots. Sera were collected prior to the first vaccination and at the endpoint (38–40 days post-challenge) from non-pregnant and pregnant mice. Results are expressed as the mean of RIPC (relative index per cent) compared to the respective positive control that received the empty vector only. Significant differences of IgG levels directed against *N. caninum* were detected between the vaccine control group and the positive control group (*P < *0.0140*, Mann–Whitney *U*-test), while the IgG titers of the other two groups were not significantly reduced. In pregnant mice, IgG antibody levels were significantly decreased in the combination group, the vaccine control group and the treatment control group compared to the positive control group (**P < *0.0022* for combination group, **P < *0.0012* for vaccination control group and **P < *0.0043* for treatment control group, Mann–Whitney *U*-test).

At the endpoint of the study, corresponding to 38–40 days post-challenge, sera were collected from all dams and non-pregnant mice and IgG were measured by ELISA (Figure 4). All *N. caninum* infected adult mice seroconverted (Table 1). In non-pregnant mice, IgG antibody titers were significantly lower in the vaccine control group compared to the positive control group (^*^P < *0.0140*, Mann–Whitney *U*-test). Comparing the other two groups with the positive control group, no differences in IgG levels against *N. caninum* extracts were detected. In dams, IgG antibody titers were significantly lower in all treated groups compared to the positive control group (^**^P < *0.0022* for combination group, ^**^P < *0.0012* for vaccine control group and ^**^P < *0.0043* for treatment control group, Mann–Whitney *U*-test).

## Discussion

To this day, the development and production of a vaccine against parasitic infections remains a major challenge due to the complexity of their life cycle and biology (48). This has been shown to be particularly true for neosporosis in pregnant animals, where substantial gaps in the knowledge of placental-parasite interactions, which would be essential for rational vaccine development, remain (49). The same accounts for drug development, which is, in the field of parasitology, often hampered by high costs and the relative low market return, rendering pharmaceutical companies reluctant to invest in the production of novel, safe and efficacious anti-parasitic compounds. Thus, despite substantial efforts, many interesting lead compounds established in an academic setting get stuck when it comes to finding a partner to implement them in the “real world.” In the case of neosporosis, there is currently no protective vaccine and no efficacious drug on the market.

The inherent difficulties encountered in neosporosis vaccine development has sparked the interest in drugs that could be used to prevent the loss of offspring and abortion that is caused by *N. caninum*. The focus has clearly been on drug repurposing, which has led to the identification of a variety of compounds, safe to use in pregnant animal models and efficiently limiting *N. caninum* tachyzoite proliferation *in vitro* and dissemination *in vivo* (6). Among those, BKIs that target CDPK1 appeared as a promising compound being active *in vivo* and *in vitro* against several apicomplexan parasites, including *N. caninum* (19). One of these compounds is BKI-1748, that has been shown to be safe in pregnant mice and partially effective in the pregnant neosporosis mouse model (28, 29).

Due to partial protection against neosporosis with both the vaccine and the drug arm, we tested a combined vaccine-linked chemotherapy strategy. Originally, this approach has been developed to combat cancer. Certain anti-cancer agents have immune-modulatory effects that result in up-regulation of surface expression of MHC molecules, tumor-associated antigens, or Fas on malignant cells, rendering them more susceptible to immune destruction (50). In murine studies, drugs such as cyclophosphamide, doxorubicin, paclitaxel, and docetaxel enhanced anti-tumor immune responses to a whole tumor-cell vaccine (51). Examples where such an approach has been translated into clinical application are e.g., in prostate cancer (52, 53) and small cell lung cancer (54). Chemotherapy-linked vaccine approaches have been used against other protozoan parasites employing inactivated parasite extracts or recombinant antigens. In a study carried out in mice, a first-generation antigen Killed *Leishmania donovani* (KLD) along with a standard drug sodium stibogluconate (SSG) and a newly tested anti-leishmanial cisplatin were used. Results showed that combination of drug and KLD significantly reduced the parasite burden, enhanced the delayed type hypersensitivity responses, and increased IgG2a levels and Th1 responses, as compared to mice given chemotherapy or immunotherapy alone (55). In addition, anti-leishmanial drugs such as *N*-methyl meglumine antimoniate and sodium stibogluconate were used in combination with the recombinant Leish-110f + MPL-SE vaccine in dogs, and a 78 kDa antigen with or without monophosphoryl lipid A (MPL-A) was applied in mice, respectively, also showing enhanced efficacy compared to drug or vaccine application alone (34, 35). For *T. cruzi* infection in mice, benznidazole in combination with a recombinant vaccine candidate, Tc24 C4 was shown to lead to significantly reduced blood and tissue parasite burdens (37, 38).

A combined vaccine-linked chemotherapy strategy had been tested previously using a sublethal dose of *N. caninum* tachyzoites, which induced a high level protection of both dams and offspring against re-infection. However, treatment of vaccinated mice with another CDPK1-inhibitor, BKI-1294, prior to re-infection had detrimental effects and resulted in increased vertical transmission upon re-challenge of these mice (56). This effect was attributed to a potential inactivation of the live vaccine by the drug itself. In our study, BKI-1748 evidently did not have any negative effect on the use of the *Listeria* vaccine vector. In contrary, we observed additive effects in the protection against vertical transmission of neosporosis when combining BKI-1748 with the Lm3Dx_NcSAG1 vector.

In this study, experimental infection of pregnant mice with *N. caninum* tachyzoites and BKI-1748 treatment from day 9 to 13 of pregnancy resulted in significant inhibition of postnatal mortality, resulting in a pup survival rate of 54%, which is slightly lower than the previously reported 70% by Imhof et al. (28, 29). However, in our experiment, all pups in the infection control group succumbed to infection already by day 19, while in the previous studies 95 % pup mortality was reached in 23 days (28, 29), suggesting the inoculum was more virulent in the current experiment. Similarly, in this experiment the survival rates of pups born to dams that were vaccinated with Lm3Dx_SAG1 were in the same range (50%), while in a previous study the pup survival rate for this vaccine was reported to be 67% (18). In addition, the percentage of PCR-positive pup brains in the groups undergoing single treatments were higher in this experiment (46 and 50%, respectively), compared to the previous studies, in which 41% PCR-positive pups were reported in BKI-1748 treated mice and 39% PCR-positive pups in Lm3Dx-SAG1 vaccinated mice (18). These minor but distinct variations compared to earlier studies reflect the differences in virulence that can occur with apicomplexan parasites that are passaged in culture and possibly adapt to cell culture conditions to different degrees dependent on the passage numbers and numbers of passages through mice (57). Nevertheless, the experimental group undergoing the combination treatment (vaccination and treatment following infection during pregnancy) exhibited a clearly enhanced pup survival rate of 86%, demonstrating that the combined treatment exerted profound additive protective effects regarding vertical transmission. This was also reflected in the number of PCR-positive pup brain samples in the different treatment groups, which were much lower in the group undergoing combined treatments compared to single treatment and infection control groups. Thus, combination treatment further reduces vertical transmission and pup mortality induced by experimental *N. caninum* infection in pregnant mice, compared with either intervention alone.

In terms of reducing the parasite load in the brain tissues of the dams, the combination treatment did not confer a clear benefit. Although the number of PCR positive brains was reduced to three out of six, compared to six out of six in the positive control, the overall parasite load was not significantly diminished. This in contrast to non-pregnant mice, where the combination treatment resulted in only two out of eight mice PCR positive in the brain, and the overall parasite load was significantly lower compared to the positve control group. Vaccination with Lm3Dx_SAG1 alone, however, appeared to be the most efficient means of reducing the parasite load in non-pregnant mice, which is also reflected in the significantly reduced IgG response measured in this group compared to the positive control group. In the dams, however, all three experimental treatment groups exhibited reduced IgG levels. This could reflect the overall reduced parasite load, or could mirror the ongoing immunomodulation during pregnancy. Surely further studies need to be carried out on the immunological features of this vaccine-linked chemotherapy approach.

Overall, however, the combination treatment was highly beneficial in reducing vertical transmission and pup mortality, but provided only modest impact in terms of cerebral infection in dams, and a slight benefit in non-pregnant mice. When looking at rates of neonatal mortality, and the numbers of pups per dam in each treatment group, the combination treatment did not induce adverse effects on fertility and pregnancy outcome. Thus this approach can be looked upon as a safe procedure, and should be further investigated as a strategy for preventing the detrimental effects of neosporosis. An improved multivalent vaccine combining multiple epitopes could potentially improve the efficacy of the vaccine and should be further evaluated.

In case vaccine-linked chemotherapy would be adopted in the target hosts such as cattle, sheep or goats, different strategies could be considered, especially in terms of practicality and cost-effectiveness. For instance, vaccination of all animals could be carried out at any given time point regardless of their infection status, and an additional treatment could be done at the onset of pregnancy. Other solutions could include vaccination and treatment of dams only prior to gestation, or vaccination for all and treatment only in symptomatic and/or seropositive animals. Whether any of these strategies is feasible is not yet clear and needs to be further evaluated.

In principle, vaccine-linked chemotherapy could be applied using a variety of different vaccines and drugs that affect *N. caninum* proliferation and viability. Besides BKIs, other compound classes should be evaluated, including endochin-like quinolones (ELQs), for which profound *in vivo* activities in the pregnant neosporosis mouse model have been recently reported, also in combination with BKI-1748 (28, 29). Other suitable candidate drugs include buparvaquone (58), artemisinin derivatives (59), and potentially also pentamidine derivatives such as Di-cationic arylimidamides (60). In any case, these compounds must be safe for use in pregnancy. Possibly also different types of vaccines could be very useful, provided they are able to elicit reasonable protection against vertical transmission in pregnant animals *per se*. This includes either viral or bacterial expression systems, or recombinant antigens. One candidate could be a polyvalent vaccine formulation composed of bacterially produced OprI-NcPDI, OprI-NcROP2 and OprI-NcROP40 (O-Ags), which was shown to induce a mixed Th1/Th2 immune response in adult mice, significantly increased protection against cerebral infection, and reduced vertical transmission and postnatal disease in offspring (42).

## Conclusions

The results of this study suggest that a vaccine-linked chemotherapy approach could be potential alternative strategy to fight infections by *N. caninum*. How this strategy could be implemented needs to be assessed in further studies. In any case, this approach may also be potentially applicable to other related apicomplexan parasites of veterinary and human importance. In addition, vaccine-linked chemotherapy can contribute to overcoming risks of drug resistance and toxicity issues by reducing drug dosage or treatment duration.

## Data Availability Statement

The original contributions presented in the study are included in the article/supplementary material, further inquiries can be directed to the corresponding authors.

## Ethics Statement

The animal study was reviewed and approved by Veterinärdienst des Kantons BE, Sekretariat Tierversuche, Herrengasse 1, 3011 Bern.

## Author Contributions

The study was conceived and designed by AH, DI, WP, and AO. Design and cultivation of the Lm3Dx and Lm3Dx_SAG1 vector was carried out by WP, CM, and AO. BKI-1748 was provided by WV and KO. The *Neospora* NcSpain-7 isolate was provided by L-MO-M. Cell culture of *N. caninum* tachyzoites and the experimental work in the mouse model was carried out by DI, CS, and WP. Antibody measurements were done by DI and CS. Interpretation of the results was done by DI, WP, CS, AH, and AO. The initial draft of the manuscript was written by DI and CS. The paper was revised by AH and DI. Final revision was carried out by all authors.

## Funding

This work was funded by the Swiss National Science Foundation grant No. 310030_184662, the National Institutes of Health NIH, grant numbers 1R01AI155412-01 and R01HD102487, and the USDA/NIFA grant number 2019-07512. The APC was funded by the Swiss National Science Foundation.

## Conflict of Interest

WV was an owner/officer of ParaTheraTech Inc, a company which was seeking to bring bumped kinase inhibitors to the animal health market. The remaining authors declare that the research was conducted in the absence of any commercial or financial relationships that could be construed as a potential conflictof interest.

## Publisher's Note

All claims expressed in this article are solely those of the authors and do not necessarily represent those of their affiliated organizations, or those of the publisher, the editors and the reviewers. Any product that may be evaluated in this article, or claim that may be made by its manufacturer, is not guaranteed or endorsed by the publisher.
